# Practices and Perspectives on Latrine Use, Child Feces Disposal, and Clean Play Environments in Western Kenya

**DOI:** 10.4269/ajtmh.19-0389

**Published:** 2020-03-02

**Authors:** Anna Ellis, Emilie E. McClintic, Emily O. Awino, Bethany A. Caruso, Kimberly R. J. Arriola, Sandra Gomez Ventura, Alysse J. Kowalski, Molly Linabarger, Breanna K. Wodnik, Amy Webb-Girard, Richard Muga, Matthew C. Freeman

**Affiliations:** 1 Department of Environmental Health, Rollins School of Public Health, Emory University, Atlanta, Georgia;; 2 Department of Behavioral Sciences and Health Education, Rollins School of Public Health, Emory University, Atlanta, Georgia;; 3 Nutrition and Health Sciences, Laney Graduate School, Emory University, Atlanta, Georgia;; 4 Hubert Department of Global Health, Rollins School of Public Health, Emory University, Atlanta, Georgia;; 5 Uzima University College, Kisumu, Kenya

## Abstract

Exposure to fecal pathogens contributes to childhood diarrhea and stunting, causing harmful short- and long-term impacts to health. Understanding pathways of child fecal exposure and nutritional deficiencies is critical to informing interventions to reduce stunting. Our aim was to explore determinants of latrine use, disposal of child feces, and perceptions and provisions of a safe and clean child play environment among families with children under two (CU2) years to inform the design of a behavior change intervention to address water, sanitation, and hygiene (WASH), and nutrition behaviors. In 2016, we conducted a mixed-methods formative research in western Kenya. We conducted 29 key informant interviews with community leaders, health workers, and project staff; 18 focus group discussions with caregivers of CU2 years; and 24 semi-structured household observations of feeding, hygiene, and sanitation behaviors. We used the capability, opportunity, motivation, and behavior model as our theoretical framework to map caregiver behavioral determinants. Latrine use barriers were lack of latrines, affordability of lasting materials, and social acceptability of unobserved open defecation. Barriers to safe disposal of child feces were lack of latrines, time associated with safe disposal practices, beliefs that infant feces were not harmful, and not knowing where children had defecated. Primary barriers of clean play environments were associated with creating and maintaining play spaces, and shared human and animal compounds. The immediate cost to practicing behaviors was perceived as greater than the long-term potential benefits. Intervention design must address these barriers and emphasize facilitators to enable optimal WASH behaviors in this context.

## INTRODUCTION

Exposure to fecal pathogens early in life results in both acute and chronic morbidity. Diarrheal diseases account for 446,000 deaths of children younger than 5 years annually.^1^ Growth failure, manifesting as stunting, results from the interactions of chronic undernutrition with acute and repeated infection with fecal pathogens.^2^ Stunting affects 24% of children younger than five years, mostly those from low- and middle-income countries (LMICs).^2,3^ Environmental enteric dysfunction (EED), an established risk factor for growth impairment via malabsorption, is almost universally found in places where water, sanitation, and hygiene (WASH) are suboptimal and enteric pathogen carriage is high.^4^ Undernutrition and infection resulting in growth failure in early childhood have long-term impacts, including impaired motor and cognitive development, reduced schooling performance, and lower economic productivity in adulthood.^5–7^

Designing interventions that sufficiently reduce fecal pathogen exposure to impact growth and diarrhea has proved a challenge. Three impact evaluations of WASH and nutrition interventions found mixed impact on diarrhea and no impact on stunting.^8–11^ Recent findings of the WASH Benefits trials in Bangladesh and Kenya demonstrate that the interventions may not have been sufficiently intensive to block fecal transmission or that they were not the appropriate interventions to address all fecal pathways.^12^ However, the Sanitation Hygiene Infant Nutrition Efficacy (SHINE) trial in Zimbabwe included a previously neglected pathway: exposure within the household living compound; they found that although the intervention reduced behaviors (geophagia and ingestion of chicken feces), it did not result in the complete prevention of these activities.^11^

Infants and young children in LMICs may be exposed to zoonotic and human pathogens through their immediate play environment within the household compound, typically a dirt-packed yard. Zoonotic pathogens from animal stool, commonly found in shared human and animal environments, may be major contributors to the global burden of enteric disease.^13^ In Zimbabwe, young children who crawled or played on dirt floors or compounds were exposed to *Escherichia coli* through ingestion of soil and chicken feces.^14^ In Bangladesh, children who ingested soil through daily activities were shown to have an elevated risk of stunting.^15^ Studies have shown that children’s feces contain a higher enteric load than adults,^16^ and the potential for household contamination is likely compounded by local disregard for child feces. Cleaning children post-defecation, washing children’s clothes post-defecation, and cleaning the compound may also be potential pathways of contamination with human pathogens, especially if caregivers are not washing their hands with soap after such activities.^16^

Although several recent studies have addressed the disposal of child feces and related practices,^14,17–19^ they have not applied these findings within a theoretical framework for intervention design. Research on the fecal–oral pathway of children under two (CU2) years has largely consisted of observational studies, but it has rarely combined observations with qualitative methods to understand why people may or may not practice behaviors that contribute to these pathways.^13,14,17,19^ Although we may be able to make generalizations of how to address potential pathways of fecal–oral transmission, the specific study context must be understood to create an intervention that addresses multiple pathways while being locally acceptable and feasible.^20^

We sought to understand what determinants influence caregivers’ practices of latrine use, safe child feces disposal, and caregivers’ perceptions of what a “safe” and “clean” play environment are, and how they prioritized making that space for children. Here, we detail the approach and findings of formative research to inform the development of a behavior change intervention to improve compound cleanliness in western Kenya. Our guiding theoretical framework was the capability, opportunity, motivation, and behavior (COM-B) framework,^21^ which focuses on three essential behavioral determinant domains necessary to practice a specific behavior. People must have the physical and psychological *capability* to perform the behavior, the physical and social *opportunity* to do the behavior, and the automatic and reflective *motivation* to practice the behavior over other competing priorities. These data were subsequently used to develop a theory-informed and evidence-based intervention that was tested using an experimental design to assess changes in behavior.^22,23^

## METHODS

We used an exploratory qualitative design to determine the capability, opportunity, and motivation of the following behaviors: 1) use of household latrines, 2) safe disposal of infant and young child feces, and 3) provision of protected hygienic play environment for CU2 years. To meet our aims, we conducted focus group discussions (FGDs), key informant interviews (KIIs), and household observations.

### Study site.

This research was conducted within eligible communities for inclusion within THRIVE II, a project led by Catholic Relief Services (CRS) that began programming in 2017. THRIVE II used the care group model^24^ to deliver messages through community and CRS-selected care group leaders to caregivers of HIV/AIDS-affected CU2 years to encourage early stimulation, positive parenting, and optimal WASH and nutrition behaviors. We purposively selected six communities within surrounding health facilities in Migori (*N* = 3) and Homa Bay counties (*N* = 3). Communities that had a minimum number of six women who were pregnant or had children ages two and younger and variability in the following characteristics were selected: agroecological zone, distance to the nearest health facility, and distance to the nearest urban center.

Migori and Homa Bay counties have the highest rates of diarrheal disease in Kenya at 28% and 24%, respectively. The prevalence of stunting (height-for-age Z scores-2SD) in Migori County was 26% for children younger than five years and 19% in Homa Bay County.^25^ An internal CRS survey of eligible recruits for THRIVE II Kenya found that before programming, 77% of respondents reported having some kind of latrine, 70% reported disposal of child feces in a latrine, 33% reported burying feces in the yard, 83% reported keeping poultry in their compound, 66% reported keeping other animals/livestock, and 72% reported the presence of an area where CU2 years could play that was separated from animals and other sources of fecal contamination.^26^ Our work contextualized these findings to gain deeper understanding of these behavioral determinants through direct observations of household and caregiver behavior and discussion with stakeholders.

### Study design and data collection.

Following a 2-week training, data were collected by eight data collectors from October to December 2016. All guides were piloted before use; adjustments were made based on data collectors’ experience and feedback from piloting. All activities, summarized in Table 1, were carried out in Luo, except where a preference for English or Kiswahili was stated.

**Table 1:** Research activities completed with participant groups in Migori and Homa Bay counties, October–December, 2016

Method	Population	Number of activities	Number of participants
Focus group discussions	Pregnant women and mothers of child(ren) younger than two years	12	68
	Fathers	6	36
	Grandmothers/step-grandmothers	6	36
Key informant interviews	Community health extension workers	5	5
	Catholic Relief Services/partner staff	7	7
	Religious leaders	6	6
	Community leaders	5	5
	Community health volunteers	6	6
Household observations	Household of caregiver of child(ren) younger than two years	24	12
Total		71	149

#### Key informant interviews.

We conducted KIIs with religious and community leaders, and CRS and implementing partner staff to determine their understanding of the current capability, motivation, and opportunity factors that influence the uptake of practices surrounding WASH, and also assess factors that could influence intervention implementation. Community and religious leaders were selected by CRS staff based on their knowledge and experience working in the study areas.

#### Focus group discussions.

Focus group discussions were held in local churches and health units with mothers and fathers of pregnant women, and grandmothers of CU2 years to understand practices related to sanitation, child feces disposal, and environmental hygiene. Pregnant women and mothers were selected based on their participation in THRIVE II; grandmothers and fathers were related to the THRIVE II participants. Focus group discussions with mothers and grandmothers were conducted by women; FGDs with fathers were conducted by men.

#### Household observations.

In each of the six communities, we targeted one household with an index child between the ages of 6–12 months and one household with an index child between the ages of 13–24 months for semi-structured observation for a total of 12 households. Participating households had a female caregiver participating in THRIVE II who had agreed to the observation. Targeting caregivers with children of different ages enabled observation of potential differences in hygiene behaviors. The focus of observation was the index child.

We conducted two observations of the same households over two consecutive days, the first day for 4 hours and the second day for 6 hours, for a total of 119.25 hours across the 12 households. Observations were conducted between the hours of 09:00 and 16:00; 09:00 was the earliest time that care group volunteers would accompany research assistants to households. To reduce potential reactivity bias, we returned to households over 2 days in hopes of increased caregiver comfort in the presence of the observer.

Research assistants used an author-developed semi-structured observation tool to record the following behaviors related to safe compounds: 1) child defecation, including where the child defecated, whether they were wearing any clothing, whether feces were cleaned up and when, who cleaned up, when and where feces were disposed, whether children were washed, how water used for washing children or clothing was disposed; and 2) child play environment, including where the child played in the compound, and whether they moved about, animals or poultry were present, children came in contact with animals throughout the day, animal/poultry feces were present, human feces were present, anyone played with the child, the child was placed on a mat, the child put anything in their mouth, and how children were monitored.

On the second day, research assistants used a structured observation tool to collect information about the compound’s environmental hygiene and the presence, functionality, and use of a latrine. Research assistants recorded the types of animals and poultry present, and the presence of animal or human feces.

### Data management and analysis.

All FGDs and KIIs were digitally audio-recorded; recordings were transcribed verbatim in Luo and translated to English. Demographic and checklist data were entered into Excel. Detailed notes from household observations were written up in English. All transcripts and data files were de-identified before analysis. All documents were password-protected.

After each day of data collection, transcription, and translation, the research team discussed barriers and facilitators to the behaviors of interest; written discussion notes were shared with the research team. We used thematic analysis^27,28^ to identify common thematic behavioral determinants, preventing or facilitating each behavior, and developed a priori codes, including those aligned with the behavioral determinants identified in the COM-B framework.^29^ This framework categorizes the determinants shaping behavior into different categories (capability, opportunity, and motivation) and six subcategories (physical and psychological capability, physical and social opportunity, and reflective and automatic motivation) (Table 2). Researchers coded transcripts in MaxQDA v12 and met weekly to discuss iterations to the codebook and ensure that they were coding in sync. Researchers wrote memos in a table format to triangulate common determinants across locations and participant type. Observation data were reviewed and entered into an Excel document organized by the codebook.

**Table 2:** Capability, opportunity, motivation, and behavior definitions^21^

Behavioral determinant	Definition	Behavioral subcomponent	Definition
Capability	Individual’s physical and psychological capacity to engage in the activity concerned. This includes having the necessary knowledge and skills	Psychological	Capacity to engage in the necessary thought processes—comprehension and reasoning
		Physical	Having physical skills, strength, and stamina
Opportunity	Factors that lie outside the individual that make the behavior possible or prompt it, including time, physical access, affordability, and social acceptability	Social	Including interpersonal influences, social cues, and cultural norms
		Physical	What the environment allows or facilitates in terms of time, triggers, resources, locations, and physical barriers
Motivation	All brain processes that energize and direct behavior, not just goals and conscious decision-making	Reflective	Self-conscious planning and evaluations, and intention
		Automatic	Processes involving wants and needs, desires, impulses, and reflex responses

### Ethical approval.

The research protocol was reviewed and approved by Emory University’s Institutional Review Board (Atlanta, GA) (#IRB00090057), the Great Lakes University of Kenya Research Ethic Committee (Kisumu, Kenya) (#CREC/1954/2017), and the Government of Kenya National Commission for Science, Technology and Innovation (Nairobi, Kenya) (NACOSTI/P/16/72200/13631). All participants provided written informed consent.

## RESULTS

We conducted 24 FGDs with mothers (*N* = 12), fathers (*N* = 6), and grandmothers (*N* = 6); 29 KIIs with community stakeholders; and 24 household observations in 12 households. Here, we present demographic information of the population engaged in FGDs (Table 3). We discuss the key behaviors of study: latrine use and child feces disposal, and the participant-identified determinants of a safe and clean play environment. We present a table of these determinants of latrine use, child feces disposal, and prioritization of a safe and clean child play environment into a table connecting each determinant (whether a facilitator [+] or a barrier [−]) to the relevant domain within COM-B (Table 4).

**Table 3:** Demographic data of focus group discussion participants

	Mothers	Fathers	Grandmothers
Characteristic	Overall (*n* = 68)*	Overall (*n* = 36)*	Overall (*n* = 36)*
Age (years)	28 (18–45)	38 (25–68)	56 (25–87)
Number of children	4	4.5	7
Number of people in household	6	7.5	5.5
Number of people in compound	8.5	8	10
Education, n (%)			
Some primary school	48 (71)	17 (47)	32 (92)
Some secondary school	16 (22)	14 (39)	2 (6)
Some tertiary school	4 (7)	5 (14)	1 (3)
Occupation, n (%)			
Business	28 (41)	7 (19)	7 (20)
Homemaker	21 (31)	–	5 (14)
Fishing	–	5 (14	–
Farmer	8 (12)	13 (36)	21 (60)
Other	6 (16)	9 (25)	2 (6)
Sanitation access, n (%)			
Can access a latrine†	43 (63)	24 (67)	25 (71)
Primary water source, n (%)			
River/lake/pond/stream	31 (46)	15 (42)	18 (51)
Piped water	18 (27)	7 (19)	7 (20)
Water pan	6 (9)	4 (11)	4 (11)
Deep bore hole	9 (13)	4 (11)	1 (3)
Other	4 (5)	1 (14)	5 (15)

* Numbers are mean or number (percent).

† Owned, shared, neighbor, or public.

**Table 4:** Capability, opportunity, motivation, and behavior (COM-B) enablers (+) and barriers (−) mapped to latrine use, child feces disposal in latrine, and provision of safe play environment

COM-B Behavioral determinant definition	Latrine use	Child feces disposal in latrine	Provision of “safe” play environment
Physical capability (physical skills, stamina, and strength)	(+/−) Physical ability to access latrine	(+/−) Physical ability to access latrine	
	(−) Some caretakers lacked control of defecation	(−) Infants lacked the ability to control defecation	
Psychological capability (knowledge, skills, and behavioral regulation)	(+) Caretakers believed exposure to human feces could cause disease to humans and animals	(+) Children trained to defecate in specific place (caretakers know where to look for feces to clean)	(−) Caretakers believed animal feces are harmful to humans
	(−) Caretakers lacked skills to build long-lasting latrine	(−) Caretakers believed that infant feces were not harmful	
Physical opportunity (environmental context and resources, including time, affordability of resources, access, and enabling environment)	(−) Caretakers lacked latrines	(−/+) Tools for disposal depended on what was accessible/available	(+) Infants can be placed in one area
	(−) Caretakers lacked funds for construction	(−) Caretakers lacked latrines (see physical opportunity)	(−) Affordability of “play” area
	(−) Caretakers lacked funds for materials	(−) Caretakers lacked access to water to wash clothing or clothes used as diapers	(−) Caretaker responsibilities/time prevented them from watching young children
	(−) Physical environment prevented lasting latrines—loose sandy soil, rocky soil, frequent flooding during the rainy season	(−) Night was not safe for caretakers to go to latrines alone, especially women	(−) Older sibling supervision exposed children to environment outside compound
	(−) Latrines smelled bad	(−) Latrines were not close to household structures	(−)Compounds lack space where children are separate from chickens/animals
	(−) Work location (lake and field) often lacked latrine presence		
	(−) Night was not safe for caretakers to go to latrines alone, especially women		
Social opportunity (social and cultural norms, and interpersonal influence)	(+) Stigma of known/observed open defecation	(+) Social norm to maintain “clean compound” (void of visible human feces)	(+) Social norm for all family members to observe crawling/walking child as able
	(−) Social norm of unobserved open defecation	(−) Social norm to dispose of water where convenient	(+) Social norm to maintain “clean compound” (void of visible human feces)
	(−) Women lacked decision-making power to allocate funding for latrines	(−) Caretakers perceived child feces disposal to be women’s work	(−) Social norm for children to eat dirt
	(−) Single mothers and widows lacked funding for latrines	(−) Older sibling care of infant/young child resulted in feces left in open or poorly buried in compound	(−) Social norm for older siblings to supervise once children could walk
	(−) Father/daughter-in-law unable to use the same latrine (norm)	(−) Social norm to bury feces	(−) Social norm for family members to share living space with animals
Automatic motivation (wants, needs, automatic responses, and impulses)	(−) Preference of defecation outside (especially by older people)	(+) Disgust triggered by feces that smelled, specifically from children who were no longer exclusively breastfeeding and thus eating some solid food	(+) Caretakers wanted their children to have a safe and clean place
			(−) Children ate dirt and mouthed objects
	(−) Shame associated with known latrine use		(−) Children were unlikely to stay in one space if walking or crawling
Reflective motivation (self-conscious planning and evaluation, and beliefs about what is good or bad)	(+) Exposure to feces could cause disease; more extreme consequences of disease (typhoid and cholera) spurred more latrine use	(+) Judgment related to having unclean compound	(+) Fear of immediate threats to child related to unsafe/unclean spaces
			(+) Female caretakers sweep compound to create clean and safe spaces
			(+) Association between geophagy and soil transmitted helminths
		(+) Perceived negative consequences if animals consumed child feces	(−) Lack of self-efficacy to provide perceived “play space”
	(+) Fines or legal action if latrine was not built	(−) Disposal at night exposed caretakers, especially women, to dangers	(−) Lack of perceived negative consequences to children if exposed to animal feces
	(+) Judgment from guests and leaders if latrine was not built	(−)Perceived lack of risk from infant/child feces	(−) Competing interests for financial investment

### Compound characteristics.

A typical compound in Homa Bay and Migori counties had at least two structures, one primary family home and one cookhouse or other family member home; some had a latrine. Most houses were semi-permanent structure with roofs made of iron sheeting and walls made of mud, plastered over with cow dung, or a combination of cement and sand. Floors were made of mud or a combination of cement, sand, and concrete. Compounds were demarcated by shrubs or fences. Compound ground, saved for kitchen gardens, was often hard-packed from frequent use by animals and humans.

### Latrine practices.

Many participants believed the time–cost to construct basic pit latrines and the financial burden to build “long-lasting” latrines were too high, affecting latrine presence. The physical environment posed challenges related to construction and sustainability of latrine structures; participants described soil that was loose and sandy, or rocky. In the rainy season, flash flooding was common; pit latrines would get washed out, and holes would collapse. Fathers reported that they lacked the skills to build “long-lasting” latrines, where pits were lined with cement. Materials for long-lasting latrines or hiring experts for construction was cost prohibitive.

Sometimes you wish to make your latrine, and when you think of how the soil is...Sometimes you have made it and covered it but when the rain comes it collapses. You give up. If I had some money then I would get cement, even two bags or three and then I construct a better latrine that can help me for four or five years. (Mother)

Latrine presence was often motivated by fear of potential negative consequences, including social stigma, health impacts, and loss of assets. Caretakers feared judgment from visitors, or fines or legal action from government workers or community leaders.

In the past, people mostly used the bushes but since the government came to the community and passed information that they will be convicting people, people with no latrine, people constructed latrines so that most people are going to the latrine. (Father)

Some also build the latrine because they were afraid they would be fined-it is just politics that people are making it. (Mother)

Caregivers stated that latrines could mitigate exposure to human feces, causing disease. Participants identified health impacts related to open defecation or exposure to human feces as diarrhea, cholera, soil-transmitted helminths, typhoid, schistosomiasis, stomach upset, and vomiting. When there was a cholera or typhoid outbreak, latrine use increased. Several caregivers were concerned that exposure to human feces could cause chicken or cows, important assets, to fall ill or die.

We have diseases, like cholera. If you don’t use the latrine, it’s going to spread so we need everybody in the house to use the latrine. It even leads to the spread of diseases among poultry within the home, a person maybe has diarrhea and if the chicken feeds on the feces, this disease can kill all the chickens within a home. (Religious leader and father)

Although participants identified some stigma against open defecation, they also stated that latrine presence, location, and condition; social norms and beliefs; season; time of day; and preference often determined latrine use. Many people lacked access to latrines: latrines were not present in their compound, near fields where crops were planted, or while fishing in Lake Victoria. Other people practiced open defecation because they preferred to be outside where the air was clean, as opposed to inside a closed, smelly latrine. Participants believed that latrines use risked immediate exposure to feces, as opposed to open defecation. In some areas, women were not supposed to use the same latrine as their father-in-law; this was disrespectful. Generally, any instance where other people knew that participants were defecating could cause shame. As men did not urinate in latrines, their use signaled defecation. In areas where compounds were more dispersed, people felt comfortable openly defecating because they would not be seen, or expose others to their “nakedness.” During the rainy season, latrines were more likely to be flooded, making them unusable. Rainy season also resulted in more plant cover, providing privacy for open defecation. Night safety meant people, especially women, dug a hole within the compound to bury feces. Risks associated with night were assailants, spirits, and snakes.

### Child feces disposal.

#### Infant and child feces disposal practices.

Of the observed households that lacked latrines (*N* = 6.5%), three households reported that they buried child feces, one threw over their fence, one disposed in a latrine, and one did not clean child feces. Research assistants observed three households burying feces and one throwing child feces outside the compound but no disposal of child feces in latrines. In FGDs, disposal of young child feces into a latrine was stated by most to be the best method if people had a latrine. Burial of child feces, during day and especially at night, was common; women’s safety fears related to night prevented latrine disposal. Caretakers buried infant/child feces inside and outside the compound; alternative methods were placing it in the garbage/compost pit, or disposing of it outside of the compound (unburied).

For caretakers to dispose of infant and child feces, they needed to know where children had defecated. Caregivers primarily kept infants naked, in clothes, or cloth/disposable diapers. Before children could crawl, caretakers easily knew when and where they had defecated. Once children could walk, they primarily defecated in the compound. Latrines threatened children’s safety; they could fall in or touch adult feces, leading to illness. Instead, caregivers discussed training children from 12 to 24 months to defecate in one designated location, through consistent reinforcement of carrying the child to the place, and through physical discipline when children defecated elsewhere.

A one-year-old will have known what you tell it to do. You can tell it that “you will be defecating here.” So the child will know that when it is pressed, even if it is in a different place, then it will run and come and defecate then you will clean the feces out. (Mother)

If it is a child who can say that I want to defecate, I can spread a newspaper and carry with it. But if she/he doesn’t know, then after defecating I can collect using some iron sheet. (Mother)

Materials or tools to aid in child feces disposal also depended on where children defecated. Female caretakers reported laying paper or torn leaves for “catching” feces and then disposing of the materials in the latrine or burying feces. Mothers also mentioned using “jembes” (small hoes), metal-corrugated sheets, or sticks; researchers observed caretakers using jembes and leaves for aid in feces disposal.

Participants reported low use of potties (a small plastic seat over a removable pot) for CU2 years. Although female caregivers reported that potty use made disposal into a latrine easier, this rarely outweighed the barriers of potty use. Potties required removal of child clothing, too much water for cleaning, more immediate cleaning because of smell and flies, and were unaffordable.

What makes people not use the potty? I said that it is economy…I can’t afford to buy potty. So it hinders some people to use the potty. (Mother)

### Opportunity barriers.

Time often determined how caregivers cleaned children, clothing, and diapers after CU2 years defecated. Although mothers reported that they knew they should throw water with fecal waste in the latrine (if present), they reported disposing of dirty water in the yard or over the fence because they were “lazy,” busy with other tasks, or because proper disposal could be “arduous.” Night affected practices; mothers kept a basin where they put soiled clothes or bedding. Lack of ready access to water when needed also impacted mothers’ ability to clean children, diapers, bedding, and the compound.

Sometimes there is no tap water because it was not pumped and yet you have a small child who defecates. You know you will have problems so you are forced to look for a place to put those things [soiled clothes]. I can take the soiled clothes and put in a basin because there is no way I can wash them at that time. (Mother)

### Child feces beliefs.

Caregivers believed that infant/young child feces, particularly from children who were breastfeeding, were clean and lacked the potential harm of adult feces because it did not smell the same. Therefore, infant/young child feces did not carry the same weight of disgust as adult feces; disposal thus lacked urgency.

We believe that when it comes to a child’s feces, it is clean. That is the truth of the matter. (Mother)

Even the one I am holding here is still breastfeeding. The feces do not smell, so people see that these feces are not harmful, they cannot bring diseases in someone’s life. (Mother)

However, when children transitioned from breastfeeding to eating *ugali*, porridge made from ground maize, wheat, or sorghum, the changed smell was a driver to more immediate child feces disposal. Not quickly disposing of this child feces lacked social acceptability and feelings of disgust. Flies were attracted to exposed and buried child feces, signaling an “unclean” compound, a source of shame.

I can feel bad because the feces could be for a child who has reached 2 years and the feces smell… When you throw it outside when people come or when people enter you home, you will hear “hey this place really has a very bad smell!” (Mother)

It is just the latrine so that we can keep our environment clean…Because if the feces are thrown, that smell will make flies to hover around and that place will not be clean. (Mother)

Caretakers perceived a potential threat to animal health from updisposed child feces. Concerns for animal well-being mirrored those that caregivers expressed related to adult open defecation; people feared animal and chicken disease and death from child feces consumption.

Moderator: You have kept on saying “the chicken should not eat [child feces], the chicken should not scatter it [child feces],” why do you say so? What would happen if the chicken eats?

Mother: Chickens will die.

### Safe and clean play environment.

#### Defining a safe and clean play environment.

Caregivers expressed the importance of keeping children safe while playing. When defining “clean” play environments for children, caregivers related similar terms and ideas that they used to describe “safe” environments. Generally, the term “safe” was defined as absence of immediate potential harms to a child, such as thorns, bushes, sticks, tall grass, stones, holes in the ground, bodies of water, broken bottles, snakes, smoke from cooking, fire, and latrines. Latrines and water, especially standing water or runoff, were perceived to pose a threat to illness.

There is still some surface run off flowing here…Sometimes they [the children] go and mess themselves in that water in the name of playing…that water will get them sick. (Mother)

### Play locations.

They said that CU2 years should ideally play on the veranda close to the house, in areas that had been swept, or within the house. Caregivers also cited that CU2 years could play under a tree, as it would be free of unsafe aspects such as snakes or thorns. Mothers and grandmothers swept and picked up sticks as an important component of creating a clean area for play; they reported sweeping daily.

I sweep the place clean for the child so that they could not find mud to put in their mouths …after sweeping] the place clean, it remains hygienic… the children play there. (Mother)

Although caregivers acknowledged the importance of keeping a child safe during play, they cited competing priorities to providing “play areas” in compounds. Only a few recognized the term “play area,” a concept they attributed to teachings by community health volunteers. Caregivers primarily associated “play areas” with community health centers, where early childhood development (ECD) spaces had been developed with assistance from CRS. These ECD spaces had covered floors, potties, and toys; they were also often in enclosed areas. Caregivers would be present for supervision while waiting for health services, and alternative caregivers, other mothers or ECD volunteers, were present to watch children. Mothers stated that fathers would not agree to pay money for a play area because they already paid for clothing and school fees for older children. Fathers acknowledged that they may not be “capable” to provide any money to a play area because “even getting enough to eat in the house is hard.” Mothers also stated that contributing money to create a play space was not a sound investment because children would lose interest or destroy it. Crawling or walking children would still require supervision.

Even if you create [it], you still have to watch them. (Mother)

### Differential needs and risks: Mobility, age, gender, and caretaker.

Caregivers differentiated the challenges of providing a safe and clean space for children depending on their physical mobility; they considered their mobility to influence their safety. Younger children who were not yet crawling or walking could be placed in a spot where the perceived risk was low. A researcher observed a mother placing her infant on a clean piece of fabric outside. Mothers were also observed placing infants on the couches inside their homes.

It depends with the age of the child, there are some who just sit, we can lay down some mat in the house for them to play on. (Mother)

Mothers identified boys, and children who could crawl, needing more parental oversight. Mothers stated that it was easier to care for young girls because they were more likely to be happy in the house, compared with boys who desired to play in the yard. Caregivers described children who were crawling (around 6 months in age depending on the child) as eating dirt and mouthing any accessible objects. Caregivers also associated this age with an increased risk of illness, and/or especially soil-transmitted helminth infection; however, worms were believed to be normative for CU2 years, and deworming pills were usually accessible through community health workers.

So you have to look at the health of the baby when she reaches the sixth month because that is where it has hard work. It sits, picks anything, even soil and eats if you place it [the baby] badly… Where the baby touches he can eat anything. (Grandmother)

During that time when the child is crawling, when they can eat soil they can get worms. (Mother)

Children closer to the age of 2 years were often cared for by older siblings. These children were more likely to leave the compound and play in fields, which caregivers said could have feces or previously defined unsafe and unclean characteristics. Caregivers cited children’s increased independence and exposure to other children as a barrier to cleanliness and safety.

### Animal feces beliefs and practices.

Caregivers did not identify the presence of animal feces as a barrier to a safe or clean environment for their children. Observations of compounds showed that 11 households had chickens. In all 11 households, chickens were unrestrained, and chickens/animals were free to go in and out of the house. In all 12 compound yards, animal and chicken feces were present, and in only three compounds was the child’s play area free from visible animal/chicken and human feces. In 24 observations, caregivers were observed sweeping the compound only four times; at no time was animal/chicken feces targeted for disposal. In two observations, children were removed from areas with animal feces when they were crawling near it; in no cases was animal feces cleaned or removed.

### Mapping to the COM-B framework.

#### Capability.

Across the behaviors, capability was not a primary determinant in caregiver behavior (Table 4). Physical capability was only related to the inability of the elderly and infirm to access and use latrines. Psychological capability was related to skills lacking to build long-lasting latrines, and caretaker knowledge related to dangers of feces exposure. Although caretakers identified adult and young child feces (older than 6 months) as being potentially harmful to animals and humans, they did not identify infant feces or animal/chicken feces as posing a risk to human health.

#### Opportunity.

Across all behaviors, determinants were mostly unsupportive for physical opportunity. In FGDs, caregivers defined many barriers to the construction, upkeep, and use of latrines, including a hospitable environment, affordability of and access to materials, and lack of skills needed to build a long-lasting latrine. Time of day/year affected adult latrine use and child feces disposal in latrines. The lack of water access affected the ability to wash materials related to infant and child defecation.

Social norms supportive of optimal behaviors related to the outward show of a practice (removal of visible child feces in compound, not being seen openly defecating). Potential judgment from neighbors, community leaders, and community health workers drove these practices; caretakers were afraid of being perceived as unclean or without shame. However, all stakeholders acknowledged that physical opportunity challenges often determined practices, demonstrating the relative weakness of these social norms.

#### Motivation.

Automatic motivation did not generally support optimal caregiver behaviors, and where it did support these behaviors, barriers related to physical and social opportunity often prevented their practice. Our research reveals that caregivers’ concerns with a safe and clean play environment stem from automatic motivations related to nurture and fear; they primarily related to visible harms that could have immediate repercussions to children, such as fire, sharp objects, and bodies of water. Disgust from the smell of adult feces and young child who had begun to defecate did motivate caretakers to dispose of feces, but lack of latrines and priority of different household tasks often proved a barrier to safe feces disposal. An important exception relates to the shift in caretaker behaviors when caregiver wants, like providing a safe space for their children, often conflicted with their knowledge, that is, that animal feces did not present harm to CU2 years.

Likewise, determinants related to reflective motivation were constrained by the barriers created by opportunity or capability determinants. Lack of perceived risk from infant and animal feces related to people’s knowledge and perception of consequences. Lack of affordable materials and environmental hazards prevented caregivers from latrine construction and upkeep, normalizing open defecation, and disposal of child feces where convenient. Time to complete behaviors was allocated for more critical and prescient activities.

## DISCUSSION

In this study, we used an exploratory qualitative design to understand the determinants that contributed to 1) caregiver latrine use, 2) child feces disposal practices by caregivers, and 3) caregiver prioritization of a clean play environment for CU2 years. We then applied COM-B domains to the determinants to better inform our intervention development and understand connections between determinants (Table 4). Although we identified barriers and facilitators to our behaviors of interest that corresponded to *capability* and *motivation*, the profound lack of *physical* and *social opportunities* were the most substantial barriers to practice. These barriers often prevented caretakers from practicing behaviors even when they were motivated.

Although caregivers knew that adult human feces posed a threat to their own, their children’s, or their animals’ health, they did not associate a commensurate risk from animal or infant feces, similar to other studies.^16,18^ This lack of knowledge could have long-term health impacts, as ingestion of chicken feces and soil containing chicken feces may be a major source of interic infection and be important in the etiology of environmental enteropathy.^13,30^ Observations conducted in Kisumu, Kenya, to understand oral contact events for infants aged 3–9 months showed they were likely to mouth their own hands approximately 0.4 times per hour.^31^ If children were playing or sleeping in areas where chickens or animals are kept, their hands were likely to come in contact with animal/chicken feces, and their hands were likely to end up in the mouth. Of additional concern is that caregivers and a community health worker reported that children and chickens both slept in the kitchen. In Bangladeshi households where animals or chickens were corralled inside at night in the same room with children, there was a significant association to EED disease activity scores and stunting.^15,23,32^ The lack of knowledge should also impact intervention design, educating caregivers about potential negative impacts of exposure to animal and chicken feces to themselves and their children.

Caregivers were concerned about the smell of feces on unwashed cloth or potties and the potential to attract flies within the compound; these concerns align with other studies where adults expressed disgust when feces were visible.^33–35^ Although caregivers who used potties or nappies/diapers were more likely to safely dispose of young child feces, this also correlated with the presence of household latrines and water: in both India and Cambodia, this meant piped water in the compound.^33,35,36^ In our study, compounds lacked nearby water access, so caregivers prioritized time and water, both examples of physical opportunity, limiting the number of times per day they would clean nappies and potties. Without latrines, caregivers were unable to safely dispose of child feces as per the Kenyan government standards.^37^ In addition, although caretakers did express interest in potties, most cited affordability as a barrier to potty use for children similar to studies conducted in Bangladesh.^35,38^ However, in Bangladesh, low-cost redesigned hoes proved to be a useful tool for removing child feces from the household compound and lacked the same cost barrier as a potty, a consideration for future intervention design.

Although parents associated cleanliness with swept dirt compounds, they were relatively inured to the social norm of children eating dirt and mouthing objects after they became ambulatory, an attitude also found in Zimbabwe. Mothers and grandmothers in Zimbabwe were unconcerned with geophagy, believing that it could benefit the child.^20^ In rural Kenya, geophagy may be a social norm; in a 1998 study, 77% of children aged 10–18 years in a sample of 203 reported eating soil daily, a risk factor for soil-transmitted helminth infections.^39^ Although caregivers in our study did not identify geophagy as a beneficial behavior, they did associate geophagy with soil-transmitted helminth infections, showing an understanding of potential consequences. However, caregivers noted that they were able to access deworming pills for children at health clinics or from community health workers. Thus, the time cost of accessing deworming pills to correct the negative outcome (soil-transmitted helminths) was relatively low, whereas the time cost to constantly observe the child was high.

Social norms were one of the most influential aspects in determining caregiver behavior and were tied to the challenges caregivers experienced related to physical opportunities such as time, affordability of materials, and access to water. Practices shifted depending on the caretaker; when older siblings were responsible, they were less likely to dispose off child feces in latrines or ensure that the area children were playing in was “safe” or clean, as found in Cambodia.^33^ Practices did not shift over the 2 days of observation; participants showed limited reactivity. Addressing social norms has been found to be critical to behavior change related to hygiene, and sanitation.^22,40–43^ Although shifting social norms forms a significant challenge, it must be considered in intervention design.

## LIMITATIONS

A limitation of this study was that the observation schedule of 09:00–16:00, selected because of the availability of female community health volunteers, our introduction into the communities, limited adult defecation observations. In addition, some observations were shortened when caretakers left the households for work or other household tasks.

## CONCLUSION

Our research revealed that the determinants of optimal caregiver practices contributing to a clean compound were myriad and were reflected through combinations of the behavioral determinants capability, opportunity, and motivation. In many WASH interventions, the theory of change, if fully articulated at all, is not typically based on rigorous formative research and behavioral theory; this is one potential reason for limited behavior change in the sector.^44^ Several recent studies have conducted formative research using predefined behavior change theories; this work reflects the growing need to identify specific determinants to challenges and develop interventions addressing those determinants.^45–47^ Categorizing the determinants using a predefined change theory supports the development of a theory-informed intervention.^23,46–49^ Caregiver motivation to practice behaviors was greatly impacted by physical opportunity, including time, physical environment, and resource scarcity and affordability. Social norms or opportunity also greatly influenced caregiver practices, contributing to unhygienic compound environments. Opportunity challenges outside the control of the program are many: the physical environment, the lack of time to practice optimal behaviors, competing priorities for caregivers, and social norms of open defecation, children eating dirt, and sharing living space with poultry and animals. A behavior change intervention designed to address these challenges will require awareness of the opportunity challenges impacting caregiver motivation and the limitations of behavior change alone to overcome these challenges.
